# Seeing emotions in the eyes: a validated test to study individual differences in the perception of basic emotions

**DOI:** 10.1186/s41235-023-00521-x

**Published:** 2023-11-03

**Authors:** Maria Franca, Nadia Bolognini, Marc Brysbaert

**Affiliations:** 1 Ph.D. Program in Neuroscience, School of Medicine and Surgery, University of Milano-Bicocca, Monza, Italy; 2 Department of Psychology and NeuroMI – Milan Centre for Neuroscience, University of Milano-Bicocca, Milan, Italy; 3 Laboratory of Neuropsychology, Department of Neurorehabilitation Sciences, IRCCS Istituto Auxologico Italiano, Via Mercalli 32, 20122 Milan, Italy; 4 Department of Experimental Psychology, Ghent University, H. Dunantlaan 2, 9000 Ghent, Belgium

**Keywords:** Emotion perception, Face mask, Social cognition, Emotional intelligence

## Abstract

People are able to perceive emotions in the eyes of others and can therefore see emotions when individuals wear face masks. Research has been hampered by the lack of a good test to measure basic emotions in the eyes. In two studies respectively with 358 and 200 participants, we developed a test to see anger, disgust, fear, happiness, sadness and surprise in images of eyes. Each emotion is measured with 8 stimuli (4 male actors and 4 female actors), matched in terms of difficulty and item discrimination. Participants reliably differed in their performance on the Seeing Emotions in the Eyes test (SEE-48). The test correlated well not only with Reading the Mind in the Eyes Test (RMET) but also with the Situational Test of Emotion Understanding (STEU), indicating that the SEE-48 not only measures low-level perceptual skills but also broader skills of emotion perception and emotional intelligence. The test is freely available for research and clinical purposes.

## Introduction

The ability to accurately interpret facial expressions is considered an important aspect of social cognition (Beaudoin & Beauchamp, 2020; Frith, 2009; Henry et al., 2016). It affects well-being in relationships, and along with other important social cognitive skills, enables us to understand, interpret and process social information to produce appropriate behavior in relation to the situation (Adolphs, 2003; Henry et al., 2016).

From a neuroscientific perspective, the ability to recognize emotional facial features is supported by integrated and distributed neural systems involving posterior areas such as the posterior superior temporal sulcus, the inferior occipital and fusiform gyri, as well as more rostral areas such as the inferior parietal lobes, the frontal operculum, the orbitofrontal cortex, and subcortical areas such as the amygdala, insula and striatum (Haxby & Gobbini, 2011; Leppänen & Nelson, 2009).

Given the extent of the neural substrates of emotional processes, it is not surprising that there are several clinical conditions that can affect emotion recognition. Autism, for example, can have a strong impact on the development of the ability to recognize facial emotions (Lozier et al, 2014). In other conditions, emotion recognition may be compromised after appropriate development, for example after brain injury (Milders et al., 2003; Yuvaraj et al., 2013), and in neurological (Marcó García et al., 2019) or psychiatric diseases (Dalili et al., 2015; Kohler et al., 2010).

Psychologist are interested in understanding how individuals perceive and respond to facial expressions under normal and pathological conditions. There are various factors that might influence this ability which warrant further investigation, as for instance effects of gender (Abbruzzese et al., 2019; Lawrence et al., 2015), age (Abbruzzese et al., 2019; Guarnera et al., 2018; Sullivan et al., 2007) and personality (Konrath et al., 2014); the role of these factors on facial emotion perception has yet to be fully delineated.

The eyes region represents a fundamental element in emotion recognition (Baron-Cohen et al., 1997). Although full face vision allows for more accurate identification of emotions (Guarnera et al., 2018), eye-tracking studies have shown that the eyes are the facial part most often observed during emotion processing (Guo, 2012; Vassallo et al., 2009), suggesting a crucial role of the eyes in transmitting important cues that allow encoding others’ emotions and internal states.

In 2020, the world faced a reality where, because of Covid-19, it became mandatory in many countries to wear personal protective equipment, especially a face mask, to prevent infection and limit the spread of the virus. For more than a year, human communication was devoid of essential social cues coming from the lower face. People had to rely entirely on emotions conveyed through the eyes. This led to an increase in research on how well people can see emotions in the eyes of others (Blazenkova et al., 2022; Carbon & Serrano, 2021; Fitousi et al., 2021; Grahlow et al., 2022; Grenville & Dwyer, 2022; Grundmann et al., 2021; Kastendiek et al., 2022; Langbehn et al., 2022; Lau & Huckauf, 2021; Leitner et al., 2022; Parada-Fernández et al., 2022; Pazhoohi et al., 2021; Swain et al., 2022; Tsantani et al., 2022; Verroca et al., 2022; Wong & Estudillo, 2022).

For example, Swain et al (2022) asked participants to identify the six basic emotions outlined by Ekman (1992) from faces with and without masks. The authors observed reduced performance with masks, which was especially true for the emotions of disgust, fear and sadness. Anger was slightly harder to identify with face masks than without; no difference was found for happiness and surprise. In addition, participants were asked to complete the Reading the Mind in the Eyes Test (RMET; Baron-Cohen et al., 2001) and the Tromsø Social Intelligence Scale (Silvera et al., 2001). The RMET is a performance test, in which eyes are shown and participants must indicate which emotions are displayed. Although the test was initially developed as a test for theory of mind (the ability to understand other people's mental states), a meta-analysis showed that the test correlates as much with measures of emotion recognition (*r* = 0.33) as with measures of theory of mind (*r* = 0.29; Kittel et al., 2022). The Tromsø Social Intelligence Scale is a subjective estimate of social intelligence in which participants indicate on Likert scales how well they think they perform on various social tasks. Swain et al. (2022) reported that emotion perception of faces both with and without masks correlated significantly with the RMET but not with the Tromsø Social Intelligence Scale, in line with many studies showing good correlations between objective tests of emotion recognition but not with tests based on self-report (Murphy & Lilienfeld, 2019).

As with all emotion perception research, having access to good stimulus material is a challenge. This was a weakness in Swain et al (2022), who used only four faces from an existing database: two from male actors and two from female actors, showing different emotions. A low number of stimuli carries a significant risk that the findings are limited to the stimulus set used and do not generalize to other stimuli (Lewis, 2023; Westfall et al., 2014). Looking at the publications listed above, we see that Swain et al. (2022) were not an exception: Ten out of the 16 papers used 10 or fewer face stimuli. Only six studies used more faces (see Table 1). Twelve studies added masks digitally to pictures from existing databases. Two studies (Fitousi et al., 2021; Leitner et al., 2022) developed face stimuli ex novo using actors with real masks. Grenville and Dwyer (2022) used a mixed approach in which they made new face stimuli of six people with and without real masks and added a third condition in which masks were added digitally to the photos without masks.

**Table 1:** Number of stimuli used in studies investigating the effects of face masks on emotion recognition

Source	Stimuli used
Blazhenkova et al. (2022)	4 Faces from database
Carbon and Serrano (2021)	4 Faces from database
Fitousi et al. (2021)	16 New faces
Grahlow et al. (2022)	36 Faces from database
Grenville and Dwyer (2022)	6 New faces
Grundmann et al. (2021)	36 Faces from database
Kastendieck et al. (2022)	6 Videos from database
Langbehn et al. (2022)	14 Videos from database
Lau and Huckauf (2021)	8 Faces from database
Leitner et al. (2022)	2 New videos
Parada-Fernández et al. (2022)	48 Faces from database
Pazhoohi et al. (2021)	16 Faces from database
Swain et al. (2022)	4 Faces from database
Tsantani et al. (2022)	8 Faces from database
Verroca et al. (2022)	10 Faces from database
Wong and Estudillo (2022)	6 Faces from database

Other researchers used the RMET (Baron-Cohen et al., 2001) to study the impact of wearing a face mask. Because face masks mainly leave the eyes visible, performance on the RMET can be seen as a good proxy of emotion perception in masked faces. Kulke et al (2022), Ong and Liu (2022), and Trainin and Yeshurun (2021) reported that performance on the RMET was better several weeks after the introduction of mandatory mask wearing than before. Trainin and Yeshurun (2021) further reported a correlation between an individual’s tendency to look at the interlocutor’s eyes and the change in RMET performance after one month of mask wearing. They concluded that ongoing everyday experiences can lead to an enhanced capacity for reading mental and emotional states by looking into the eyes of individuals, especially for people motivated to understand the mental states of others.

The RMET has an advantage over self-selected stimuli because the test is known to have acceptable reliability. The *reliability* of a test indicates how dependable the test result is. A first way to measure reliability is through test–retest reliability. If a test measures a skill consistently, there will be a high correlation between the scores obtained in the first and second administration. For example, participants who performed poorly in the first session should also perform poorly in the second session.

A second way to measure reliability is by looking at the internal consistency of the test. If a test measures a single skill, all items will have positive intercorrelations. Measures commonly used to assess internal consistency are Cronbach's alpha and McDonald’s omega. For example, Jankowiak-Siuda et al. (2016) reported a Cronbach’s alpha equal to 0.67 for the Polish version of the RMET and a test–retest reliability of 0.89. Koo et al. (2021) found values of, respectively, 0.54 and 0.78 for the Korean version, and Vellante et al. (2013) reported values of 0.60 and 0.83 for the Italian version. Fernández-Abascal et al. (2013) reported a 1-year test–retest reliability of 0.63 for the Spanish version.

Reliability is an important element in research on individual differences because a variable cannot correlate with another variable any more than with itself (Olderbak et al., 2021). Correlational research with variables for which no reliability information is available is, therefore, a risk, because it is not possible to interpret low correlations. Indeed, these may be due to the low reliability of the variables or to the lack of correlation between them. Stimulus sets with low reliability are also suboptimal for experimental research because low reliability often comes from having too many easy and/or difficult items. Performance on such items is unlikely to change much from one condition to another. Ideally, stimulus materials should include the whole range of difficult levels, going from easy to difficult. This is hard to achieve if researchers have to assemble the stimulus set themselves (as happened in the studies of Table 1).

At the same time, RMET is not an ideal test to study emotion recognition. Although it measures emotion recognition (Kittel et al., 2022; Oakley et al., 2016), it was originally developed to measure theory of mind. It measures complex emotions, such as jealousy, arrogance, and irritation, which differ from the basic emotions (anger, disgust, fear, happiness, sadness, surprise) examined in much emotion recognition research. The wide range of emotions means that the RMET measures not a single factor (skill) but a group of factors (Higgins et al., 2022), as seen in the fact that the test’s internal consistency is lower than its test–retest reliability. There are also concerns that recognizing complex emotions requires a good vocabulary (Kittel et al., 2022; Pavlova & Sokolov, 2022), which limits research with less verbally proficient groups, and the low quality of the old black-and-white pictures becomes a concern.

Because of the aforementioned theoretical context and methodological problems with available tests, the aim of the current study was to develop a new test that measures how well a person can recognize basic emotions in the eyes of others. Such a test should avoid ceiling effects in healthy participants and be useful in clinical populations to detect even subtle deficits in emotion processing.

## Study 1

The key requirement for an accuracy test is to have stimuli that differ in difficulty. This is more difficult than is sometimes thought, because it is not enough to have easy and difficult items. If the easy items are achievable for everyone and the difficult items for no one, then there will be no individual differences in scores and placing participants in an easier or more difficult condition will make little difference. What is important is to find the sweet spot of items that are not too easy and not too difficult, so that they are achievable for some participants but not for others, and so that they are achievable under some conditions but not under others. Finding critical items works best if you can start from a rich database, as it is then possible to try out many stimuli in search of good ones. Such databases have recently become available through digitization and database sharing. Many databases for emotional stimuli are reviewed on KAPODI (Diconne et al., 2022). Ultimately, we decided to use the Ryerson Audio-Visual Database of Emotional Speech and Song (RAVDESS) dataset (Livingstone & Russo, 2018).

### Method

#### Participants

A total of 358 healthy adults completed the test. They included 159 Italian participants, consisting of master students of the University of Milano Bicocca (105 females; 53 males; 1 non-binary), together with 199 first-year psychology students from Ghent University (174 females; 23 males; 2 non-binary). The study was conducted following the ethical standards of the Declaration of Helsinki, and the local Ethical Committees. All participants gave their written informed consent, before taking part in the study, and received European University Credits (ECTs) for their participation.

#### Stimulus materials

The RAVDESS dataset contains clips of 24 professional actors (gender-balanced) expressing Ekman’s six basic emotions, along with a calm (neutral) version. Each expression is performed four times: with weak and strong intensity and while participants are speaking or singing, resulting in 7356 recordings. Other advantages of the dataset are that the stimuli are freely available for research, can be shared with others, and have been evaluated by independent raters. The ratings provide information on how well the emotion can be recognized. Half of the assessments were done without sound, which were particularly interesting for us. The ratings showed that not all emotions were equally easy to recognize (in descending order: disgust, anger, surprise, happiness, fear, calmness, sadness).

For each actor, we selected two expressions for each emotion: one that was recognized by about 90% of the raters and one that was recognized by about 40–60% of the raters. This ensured that the expressed emotions were real and recognizable, at least when the full video clip was watched without sound. At the same time, we hoped that the stimuli would not be too easy and more or less equally difficult for the different emotions. Due to an oversight, we forgot to include the videos of actor 23. This gave a total sample of 315 clips, because seven out of 322 combinations (23 actors × 7 emotions × 2 recognition rates) were not present in the dataset.

**Fig. 1:**
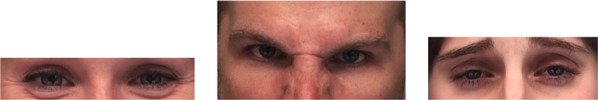
Examples of stimuli used in Study 1 showing various basic emotions

We selected what seemed to us to be the best frame from the video and cut a rectangle around the eyes. The size of the rectangle varied from item to item in a further attempt to increase variability in the stimuli (see Fig. 1).

#### Procedure

Testing happened online, based on Qualtrics software. Participants were first informed about the task and gave informed consent. Then, they were shown very clear examples of each of the 7 emotions (including full face and part of the body) together with the labels. The Italian labels were in alphabetical order: disgusto [disgust], felicità [happiness], neutrale [neutral], paura [fear], rabbia [anger], sorpresa [surprise], and tristezza [sadness]. The Dutch labels were: angst [fear], neutraal [neutral], verdriet [sadness], verrassing [surprise], vreugde [happiness], walging [disgust], and woede [anger].

After the examples, participants were told that we were investigating whether people could see emotions in eyes. They were told they would see 315 stimuli with one pair of eyes and seven labels in alphabetical order (six emotions and neutral). They had to choose the label they felt best represented the face expression. We indicated that we did not know if the task was doable, so we tried it out. Most participants found the task interesting and life-relevant.

#### Statistical analysis

We performed a psychometric analysis of the test. First, we wanted to know whether the test would reliably measure differences in performance between participants. Because we did not have two test measurements with some time in-between, we could not look at test–retest reliability, but we could measure the *internal consistency* of the test. We used the R package psych (Revelle, 2023) to calculate Cronbach’s Alpha, Omega total, and Omega hierarchical. Cronbach’s Alpha is the traditional index but makes a few assumptions that are not present in Omega total. Omega hierarchical is assumed to measure how much of the total variance is due to a single, general construct affecting all items, despite the multidimensional nature of the item set (see Flora, 2020, for a tutorial on reliability coefficients).

Second, we wanted to know whether the test measured a single factor (emotion recognition) or multiple factors. A good test is one that measures the intended trait as purely as possible. If seeing emotions in the eyes is a unitary skill, we should see that the test mainly measures a single factor. This was assessed with a *scree plot analysis* using the R package psych (Revelle, 2023). A scree plot shows the weights (eigenvalues) given to factors extracted in a factor analysis (FA) and/or a principal component analysis (PC). A test that measures a single factor is characterized by a high weight of the first factor, ideally followed by weights below 1 for the second and subsequent factors. Because the latter is not always the case, modern techniques compare the weights obtained with weights expected from a random data set with the same structure (see Sakaluk & Short, 2017, for a tutorial of exploratory factor analysis). All the figures of the scree plot analysis were taken from the R package psych (Revelle, 2023) and based on tetrachoric correlations (to take into account the binary dependent variable). A full-page version of the figures is available in the osf archive; they can also be generated from the data and the R code in the repository.

Finally, we wanted to reduce the item pool and select only items that were interesting for our test. These were items on which (1) participants with low skill scored lower than participants with high skill, (2) which came from different actors, (3) which were balanced for gender and emotion, and (4) which had varying difficult levels.

### Results

Data from Milan and Ghent labs were analyzed together, as we wanted to have a large and diverse sample to start from. No data had to be deleted because of careless responding (either random responding or repetition of the same response). Participants selected the correct response most often for happy and calm expressions, and least often for surprise and disgust (see Table 2), even though we started from equally difficult video clips (see stimulus selection). It is important to realize that the percentage correct per emotion is a combination of sensitivity and response bias. Participants who decided to press calm whenever they were in doubt, would be correct more often with calm stimuli than participants who chose to press happy whenever they were in doubt. Table 2 also shows a good standard deviation in the percentage correct per stimulus. This opens perspective for stimulus selection. Overall, participants were correct on 50% of the trials, whereas 14% was the guessing rate (100/7). All emotions were recognized above chance level.

**Table 2:** Percentage of emotions correctly chosen (guessing level = 0.14)

Emotion	Mean	SD
Anger	0.40	0.32
Calm	0.70	0.20
Disgust	0.30	0.22
Fear	0.39	0.25
Happiness	0.73	0.26
Sadness	0.65	0.22
Surprise	0.34	0.29

Mean represents the accuracy data calculated on the stimulus average. *SD* Standard Deviation

Reliability of the test (i.e., internal consistency) was very good: Cronbach’s alpha was 0.89, Omega total 0.90. And Omega hierarchical was 0.84. The high value for the Omega hierarchical, can be interpreted as suggesting that the test primarily measured a single construct (which may be subdivided into more than one part, hence the name hierarchical).

**Fig. 2:**
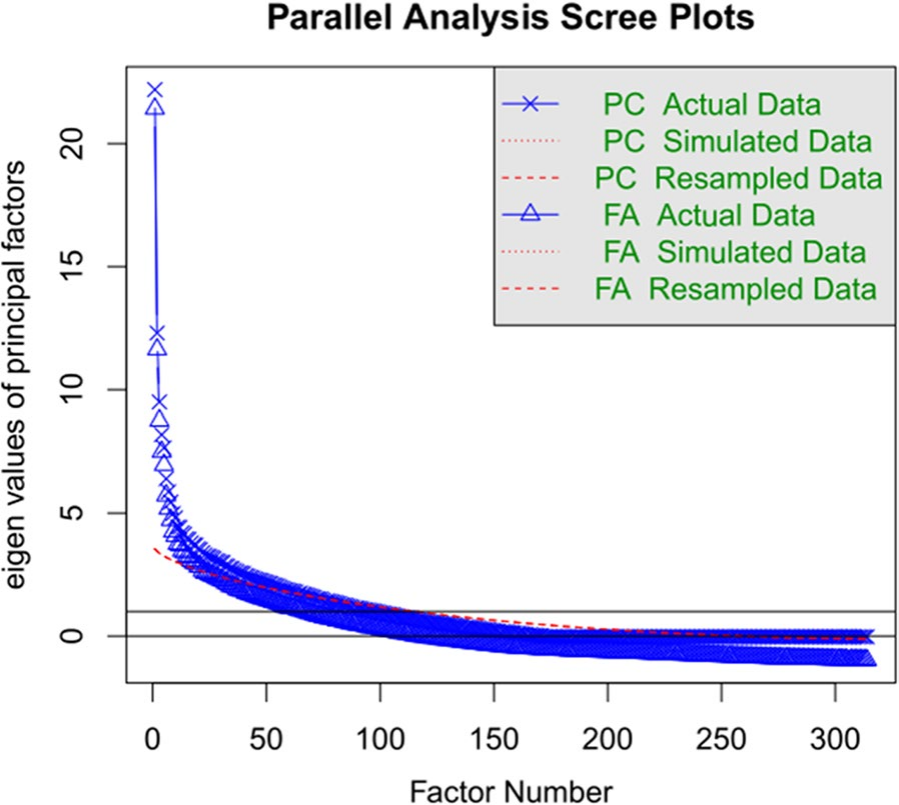
Result of a scree plot analysis of all items from Study 1. The plot shows that there are many factors with high weights (eigenvalues), which is not ideal for a test supposed to measure a specific skill

A less favorable result was found in the scree plot. As shown in Fig. 2, there were several factors with weights above 5, and more importantly, with weights considerably above those that would be expected for noise data (simulated or resampled). This indicated that the test measured multiple factors and this was true for both the outcome of a factor analysis (FA) and principal component analysis (PC). The scree plot analysis indicated that the high values of alpha and omega were not due to high intercorrelations between the items but to the large number of items in the test. The mean test result is then stable (reliable), but the test itself is influenced by many factors, even if we assume that the total test score measures a single factor (skill in emotion recognition).

Based on the outcome of the analyses, we decided to select the best stimuli per basic emotion, except for the calm (neutral) expression. The latter was dropped because it became clear that participants differed considerably in the selection of this category, introducing noise in the dataset.

The following criteria were used for item selection. First, we only selected items with an item-rest correlation higher than 0.2. Item-rest correlation refers to the correlation between performance on an item and the average performance on the other items. A good item is an item with low scores for participants who perform poorly on the other items, and high scores for participants who perform well on the other items. High item-rest correlations increase the likelihood that a single factor accounts for most of the variance. The modest item-rest correlations obtained in Study 1 indicate that there is quite a bit of measurement noise and thus that the test needs a fairly large number of items to obtain good reliability. We additionally used various IRT analyses to select items that differed in difficulty and had good discrimination power. These are not reported here but can be replicated with the R program in the osf archive.

Second, we aimed to decrease the performance differences between emotions and to increase the overall level of accuracy, as low performance is demotivating for participants.

Finally, we wanted to include as many different actors as possible and have gender-balanced lists for the various emotions.

Table 3 shows the outcome of the selection of the 60 best items. Unfortunately, it was not possible to select an equal number in each category. In particular, the number of surprise expressions was low (*N* = 6).

**Table 3:** Statistics of the 60 items chosen for validation

Emotion	Mean	SD	*N*
Anger	0.68	0.21	12
Disgust	0.59	0.17	9
Fear	0.63	0.14	11
Happiness	0.69	0.28	10
Sadness	0.73	0.15	12
Surprise	0.59	0.26	6

*SD* standard deviation, *N* number of stimuli

**Fig. 3:**
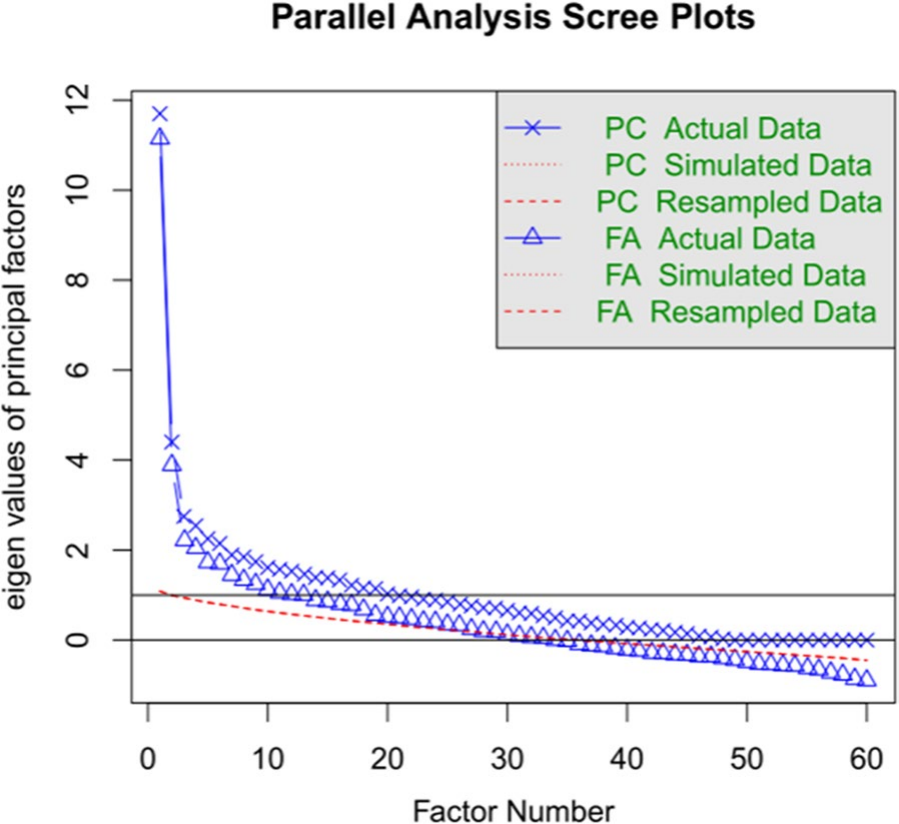
Outcome of a scree plot analysis of the 60 selected items from Study 1. This shows that the first factor had a larger impact than in the unselected sample of items

Reliability of the selected items was Cronbach’s alpha = 0.85, Omega total = 0.86, and Omega hierarchical = 0.46 (we will return to the low value of Omega hierarchical later, after Study 2). At the same time, the scree plot analysis gave stronger evidence for the importance of the first factor, which now accounted for 19% of the variance (see Fig. 3). It was still the case that the test contained several factors with eigenvalues much higher than those expected for randomly simulated or resampled data, suggesting that test performance was affected by several factors other than the one we are interested in. As reported in the introduction, the same observation has been made for the RMET, where internal consistency is lower than test–retest reliability.

### Discussion

Study 1 was a good illustration of the fact that items from a standardized database are not sufficient for a good test. Although the sample of items we took had good reliability because of its size, it proved impossible to find 12 good items for each emotion. In particular, participants had difficulties distinguishing between anger, disgust, fear, and surprise on the basis of the eyes alone (see also Swain et al., 2022). They were better at seeing happiness and sadness. They also often selected the calm (neutral) option, but we have reasons to believe this was mainly due to response bias, because the neutral option was chosen quite often for the other emotions as well. To some extent, this was an inevitable consequence of using difficult stimuli that caused accuracy differences between participants: it made participants hesitate and increased the impact of response biases. On the positive side, the data suggested that it was possible to design a reliable test.

An important caveat to the findings so far was that we could not be sure that the item selection resulted in good stimuli until we conducted a validation study on a new group of participants. At worst, it could be that the performance on the selected items was entirely due to overfitting (i.e., selecting items that happened to look good in the sample because of chance fluctuations in human performance). In that case, we would observe that the selected items in a new study would behave very similar to the unselected items of Study 1 (see Fig. 1) because of regression to the mean. We tried to avoid such a scenario by testing 358 participants from two different universities, but we could not be sure until we replicated the findings in a new sample.

Another possible danger was that our selection of items would largely measure a single factor, but that this factor would no longer correlate with emotion recognition (for example, because it reflected the perception of a visual feature, present in some faces but absent in others). The only way to find out if the selected items measured the intended skill was to see if performance on them correlated with other, established emotion recognition tests.

## Study 2

A test is only useful if the items give the same data in a new, independent sample, so that one can trust the findings. This was the first reason for running the second study.

The second reason was that we needed information on validity. If our SEE test (Seeing Emotions in the Eyes) measured emotion recognition, it should correlate well with other tests for emotion recognition (e.g., Schlegel et al., 2019). The obvious test to compare the SEE test to was the RMET (Baron-Cohen et al., 2001), given that both tests have the same format, differing mainly in the number of emotions tested (36 complex emotions vs. 6 basic emotions) and in the number of response alternatives (4 instead of 6). Another accuracy test often used to measure individual differences in emotion recognition is the Situational Test of Emotional Understanding (STEU; MacCann & Roberts, 2008). In this test, 42 social situations are verbally described and participants are asked to choose from five options the feeling that the protagonists are likely to experience.

In addition to finding convergent evidence with tests that were supposed to measure the same construct, it was informative to check to what extent the test measured related constructs. For example, it is interesting to know whether the SEE test is influenced by anxiety in participants (Demenescu et al., 2010). A widely used test to measure anxiety is the State-Trait Anxiety Inventory (STAI; Spielberger et al., 1983). This test measures anxiety both as a stable trait and in the specific test situation (anxiety as a state). A second related construct we wanted to study was apathy, a reduced motivation for purposeful activity in terms of behavior, cognition, emotion, and social functioning (Robert et al., 2018). Given the evidence that emotion perception can improve after practice (Kulke et al., 2022; Ong & Liu, 2022; Trainin & Yeshurun, 2021), it could be that performance on the SEE test is lower in people with low motivation to train emotion recognition. Apathy may also interfere with emotion recognition because it affects emotional/social dimensions, which can lead to less interest in the other person's feelings and social withdrawal. A test designed to measure apathy is the dimensional apathy scale (DAS; Radakovic & Abrahams, 2014).

All in all, we added five tests (RMET, STEU, STAI-trait, STAI-state, DAS) to the SEE, in order to better assess the value of the test.

A last reason for conducting a second study was that we were not completely satisfied with the item selection we could make based on Study 1. We had hoped to select 12 items per emotion, so that each emotion could be investigated on its own, but this proved impossible (see Table 3). Given the information we gathered in Study 1, it seemed a missed opportunity not to use the information we gained to select additional stimuli that would hopefully be clearer for surprise, fear, and disgust, and more difficult for anger, happiness, and sadness. In addition, it made sense to try to spread the number of different actors in the stimulus set as much as possible, to improve generalization to other faces. So, we added new stimuli from the faces that were not selected.

### Method

#### Participants

The participants were 200 new master students from Milano Bicocca (115 women; 85 men), who completed all tests. The study obtained ethical approval; all participants gave their written informed consent and received European University Credits (ECTs) for their participation.

#### Stimulus materials

The first set of stimuli consisted of the 60 SEE faces we selected from Study 1 (Table 3). We call this test SEE1. In addition, 72 new SEE stimuli were created to try to fill gaps. The items were distributed as follows: 10 new ones for anger, 15 for disgust, 10 for fear, 13 for happiness, 11 for sadness, and 13 for surprise. These stimuli form test SEE2. Unlike in Study 1, there were no longer neutral (calm) items, so participants had to choose between six emotionally charged options.

For RMET, we used the Italian version developed by Serafin and Surian (2004) and also used by Vellante et al. (2013) and Maddaluno et al. (2022). It contained, as the English version, one practice item and 36 test items, but the labels were translated into Italian. Each item had four response alternatives, one of which was correct.

We translated the STEU ourselves and tried to stay as close as possible to the English version. All 42 items were translated, even though it is known that not all items work well in English (Allen et al., 2014). The reasoning was that it was better to start with the largest possible pool, which could then be pruned, than to start with a reduced item set in the hope that the Italian pattern of good stimuli would follow the English one. Each item had five response alternatives, only one of which was correct.

The Italian version of the STAI was developed and tested by Pedrabissi and Santinello (1989). It contains 40 items, 20 related to anxiety in general (trait), and 20 to anxiety at the moment (state). Participants filled in a Likert scale from 1 to 4 to indicate how strongly the item applied to them.

Finally, we used the Italian version of the DAS (Santangelo et al., 2017). It contained 24 self-ratings on a 4-point Likert scale.

#### Procedure

All tests were programmed in Qualtrics and administered online. The order of the tests was the same for all participants. After explaining the purpose of the study and asking for informed consent, we first presented the 60 items of SEE1 and immediately afterward (without pause), participants saw the 72 items of SEE2. Subsequently, they were given the RMET, STEU, STAI and DAS, with the usual instructions.

#### Statistical analysis

In the second study, we focused on the validity of the test developed in Study 1 (SEE1) to examine whether it measured emotion recognition as a skill. Therefore, we investigated the convergent validity between the four tests hypothesized to measure emotion recognition (SEE1, SEE2, RMET, STEU) and to what extent these correlations differed from tests measuring other aspects of human functioning (STAI and DAS).

Next, we looked at the quality of each test, performing the same statistical procedures as in Study 1 (reliability, item analysis, factor analysis, IRT). As in Study 1, all figures from Study 2 can be reproduced using the R code and are also available as full-page figures on the osf repository.

Finally, we selected items from SEE1 and SEE2 to come to the best possible test.

### Results

As in Study 1, there was no evidence to distrust the data from participants (probably because they found the test interesting and wanted to help us make a good test).

Before going into the details of the test analysis, we had a look at the correlations between the tests. These correlations contained important validity information, because there was little to expect from item analysis if there were no significant correlations between our tests and the other tests. A comparison of the Pearson and Spearman correlations revealed that our sample included a few low and high scorers, who did not really distort the correlation pattern but still had an upward effect on the Pearson correlation. Because the elevated Pearson correlations were unlikely to be replicated in a new study, we used robust correlation calculation (pbcor; Mair & Wilcox, 2020).

We found that all four emotion recognition tasks (SEE1, SEE2, RMET, STEU) correlated strongly with each other (see Table 4). This confirms that they all measured to a large extent the same skill. The good performance of the new SEE2 items was particularly impressive, as this test was almost as good as the SEE1, even though performance on the new items was significantly lower (48% correct vs. 69% correct). Furthermore, it was interesting to see that the STAI and the DAS had positive intercorrelations, indicating that participants with high anxiety had a higher lack of motivation for goal-directed behavior. The correlations between the emotion recognition tests and DAS were negative, as expected, but not high enough to be significant. Test performance was not affected by the anxiety or apathy level of the participants (who it should be remembered were all university students).

**Table 4:** Performance on the tests (SEE1, SEE2, RMET, STEU, STAI-T, STAI-S, DAS) and correlations between the tests

	M	SD	SEE2	RMET	STEU	STAI-T	STAI-S	DAS
SEE1	0.69	0.14	**0.58**	**0.43**	**0.49**	− 0.03	0.01	− 0.12
SEE2	0.48	0.10		**0.39**	**0.44**	− 0.03	-0.04	− 0.13
RMET	0.71	0.13			**0.47**	− 0.06	− 0.09	− 0.09
STEU	0.58	0.13				0.08	0.04	− 0.01
STAI-T	2.31	0.56					**0.78**	**0.46**
STAI-S	2.16	0.57						**0.31**
DAS	2.06	0.35						

For the SEE1, SEE2, RMET, and STEU, the values of M and SD refer to percentage correct; for the STAI-T, STAI-S, and DAS the values refer to a Likert scale from 1 to 4. Correlations in bold are significant at *p* < 0.01. The other correlations are not significant at *p*  < 0.05

*M* mean, *SD* standard deviation, *RMET* reading the mind in the eyes test, *STEU* situation test of emotional understanding, *STAI-T* stait trait anxiety inventory-Trait, *STAI-S* stait trait anxiety inventory-state, *DAS* dimension apathy scale

As can be expected based on the intercorrelations, reliability was good for all tests, slightly better for the SEE tests than for RMET and STEU, in line with the wide selection of stimuli we could start from (see Table 5). A less interesting finding was that Omega hierarchical seems to be a rather unstable reliability estimate. While it was low for the SEE1 items (0.43) in Study 1, it was high (0.83) in Study 2, and at the same time it was low for SEE2 (0.21) even though that test measured the same construct in a very similar way, given the high correlation between SEE1 and SEE2. This indicates that Omega hierarchical may not be as good an indicator of test quality as has been hoped (see also Cho, 2022). The reliability of STEU was lower than 0.8 but can be improved by omitting items with a poor profile, in line with what Allen et al. (2014) did with the English test (see the osf repository for detailed item analysis and selection).

**Table 5:** Reliability of the tests

	Alpha	OmegaT	OmegaH
SEE1	0.89	0.90	0.83
SEE2	0.84	0.86	0.21
RMET	0.77	0.79	0.72
STEU	0.77	0.79	0.15
STAI-T	0.92	0.92	0.70
STAI-S	0.93	0.95	0.63
DAS	0.77	0.85	0.81

*OmegaT* omega total, *OmegaH* omega hierarchical

**Fig. 4:**
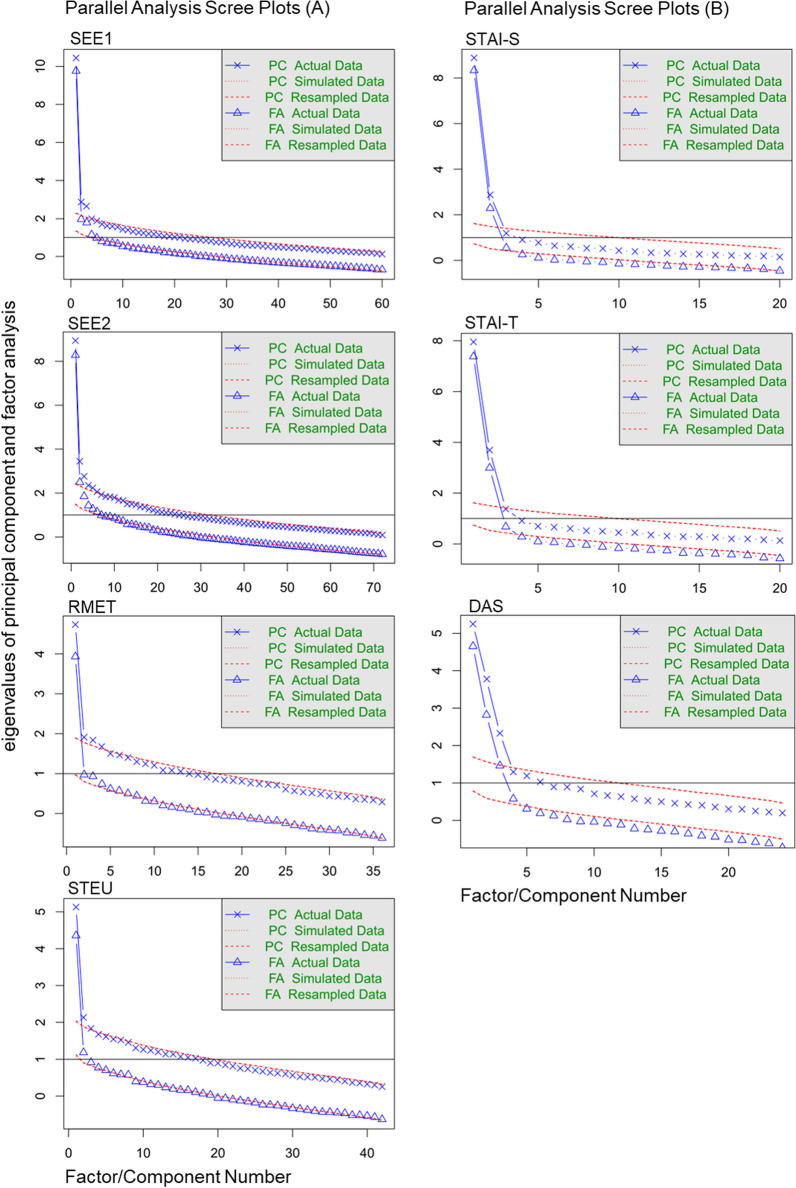
Scree plots of all tests used in Study 2. Left part: Scree plots of the four emotion recognition tests show that these tests largely measure a single factor. Right part: Scree plots show that STAI-T and STAI-S probably measure two different factors with weights considerably above those based on simulated or randomly resampled data, and that DAS may even measure three factors

Finally, Fig. 4 shows the scree plots of the seven tests. It illustrates that SEE1 did not lose much of its quality compared to Study 1 (see Fig. 3). The first factor explained much more variance than the subsequent factors. SEE2 had more features of the unselected items from Study 1 (see Fig. 1), with more evidence for the contribution of more than one factor. The scree plot further showed that the STAI was better characterized as a combination of two factors and the DAS tests as a combination of three factors, in line with previous observations (STAI: Shek, 1991; DAS: Lafond-Brina & Bonnefond, 2022; Santangelo et al., 2017). This opens up possibilities for more detailed analyses in the future, when more data are available.

### Discussion

The pattern of results in Study 2 was clearer than anticipated. The study confirmed that the four emotion recognition tests (SEE1, SEE2, RMET, STEU) largely measured the same underlying skill, even though they differed methodologically from each other and were not perfect in their details. Indeed, the RMET has been criticized several times for not being a pure measurement of a single factor (Higgins et al., 2022; Kittel et al., 2022; Olderbak et al., 2015). At the same time, it showed high test–retest reliability (Fernández-Abascal et al., 2013; Jankowiak-Siuda et al., 2016; Koo et al., 2021; Vellante et al., 2013) and good correlations with convergent tests (Kim et al., 2022; Schlegel et al., 2019; the present study). Similarly, the STEU has been criticized for containing many suboptimal items (Allen et al., 2014). Nevertheless, looking at the correlations with related tests, the STEU seems to perform well at an average level. The high intercorrelations between the tests suggest that statistical fit criteria of individual tests may be less important than sometimes thought in the context of confirmatory factor analysis (Hopwood & Donnellan, 2010) and may even lead to construct underrepresentation (Messick, 1989) if the underlying skill is hierarchically organized and the test does not systematically map the subcomponents (Neubauer & Hofer, 2022; Steger et al., 2019). Also, Omega hierarchical seems to differ too much between very similar tests or even two runs of the same test to be used as a strong cue for test quality (Cho, 2022).

**Fig. 5:**
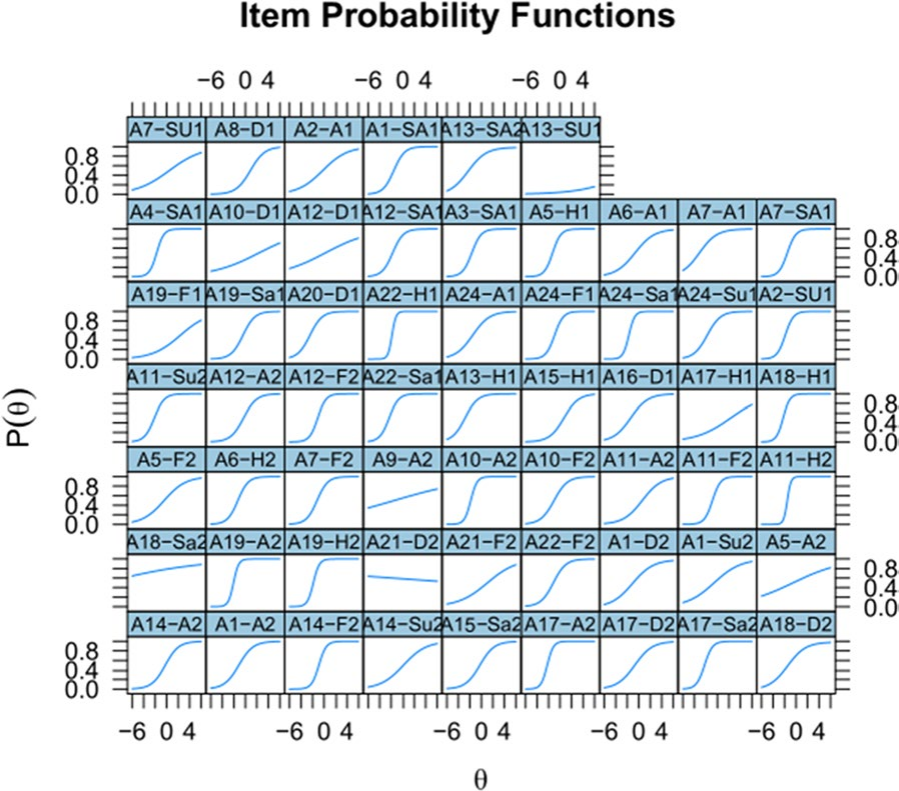
Item Response Theory analysis of SEE1 in Study 2: Most items show the expected pattern of performance (low at the left of the curve and high at the right) except for a few (e.g., A21-D2, A13-SU1, A9-A2 and A18-Sa2)

**Fig. 6:**
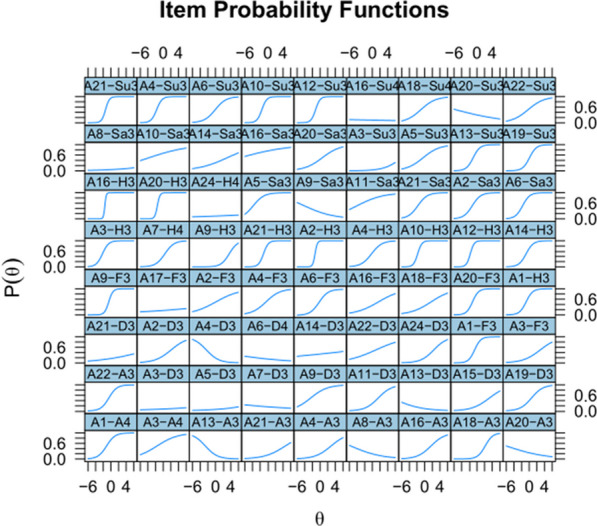
Item Response Theory analysis of SEE2, showing several bad items with higher scores for low-proficiency participants than for high-proficiency participants (e.g., A20-Su3, A9-Sa3, A4-D3) among good ones (A6-Su3, A20-Sa3, A2-H3, A20-F3, A19-D3, A22-A3)

A further interesting finding of Study 2 was that SEE2 seemed to measure the construct of interest (the ability to recognize emotions) nearly as well as SEE1 (high correlation between both tests and high correlations with the other emotion-recognition tests), even though several items were far from being optimal. The latter can be seen an in item response theory (IRT) analysis. IRT analysis provides the same information as item-rest correlation, but in more detail, because it shows how performance differs as a function of participant skill. An items is good if low-proficiency participants have a low chance of knowing the answer and high-proficiency items have a high chance. In addition, the transition from low to high is rather steep (item discrimination) and differs between items (item difficulty). The IRT analysis was conducted using the R package mirt (Chalmers, 2012). Figure 5 shows the outcome of the analysis for SEE1; Fig. 6 shows the outcome for SEE2.

Combining the items from SEE1 and SEE2, we were able to compose a good test with 48 items (SEE-48). The test contains 8 matched items for each of the emotions, coming from 22 different actors (four male and four female per emotion). Performance on the items varied from 40 to 90% with an average of 63–70% per emotion, as shown in Table 6.

**Table 6:** Accuracy rates for the SEE-48 stimuli in Study 2

Stimulus	Anger	Disgust	Fear	Happy	Sad	Surprise
S1	0.91	0.88	0.78	0.91	0.83	0.88
S2	0.87	0.74	0.77	0.80	0.83	0.87
S3	0.69	0.70	0.75	0.80	0.80	0.82
S4	0.64	0.70	0.72	0.77	0.78	0.72
S5	0.61	0.64	0.69	0.69	0.73	0.62
S6	0.56	0.60	0.56	0.68	0.59	0.54
S7	0.55	0.41	0.56	0.54	0.55	0.54
S8	0.41	0.35	0.49	0.42	0.44	0.49
**Mean**	**0**.**66**	**0**.**63**	**0**.**67**	**0**.**70**	**0**.**69**	**0**.**69**
**SD**	**0**.**17**	**0**.**17**	**0**.**11**	**0**.**16**	**0**.**15**	**0**.**16**

S1 is the easiest item for each emotion; S8 is the most difficult item for each emotion. See the osf site to find the pictures corresponding to these stimuli

**Fig. 7:**
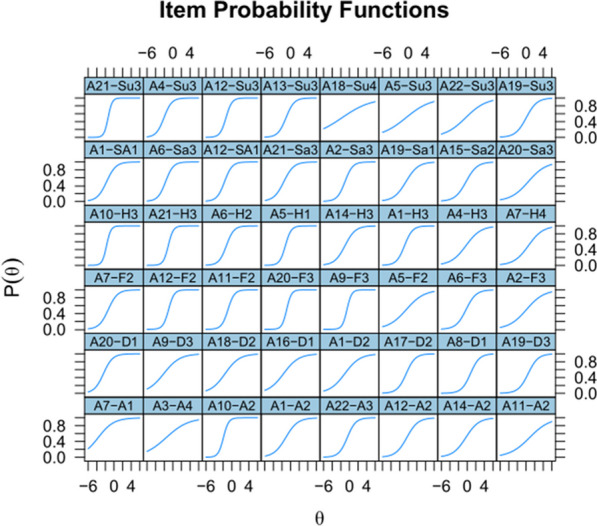
Item Response Theory analysis of the SEE-48 items

Figure 7 gives the outcome of an IRT analysis of the selected items. These provide a test with reliability of Cronbach’s alpha = 0.87 and Omega total = 0.90 (reliability is likely overestimated due to overfitting but should still be above 0.8 in a new sample, given the similarity of the data in Study 1 and Study 2). Robust correlation was 0.49 between SEE-48 and RMET, and 0.46 between SEE-48 and STEU. The negative correlations with STAI-T, STAI-S and DAS were not significant.

## General discussion

Humans are able to see emotions in the eyes of others (Allen-Walker et al., 2015; Baron-Cohen et al., 1997; Biotti & Cook, 2016). This ability underlies social interactions and well-being and has recently received attention due to the mandatory wearing of a face mask during Covid-19. Despite a considerable number of publications, we could not find a good test when we wanted to include the ability in our research. In particular, we could not find a test for basic eyes emotion recognition that contained information on reliability and validity. Therefore, we decided to create such a test, which we call SEE-48 (Seeing Emotions in Eyes, consisting of 48 items). Making the test was easier than expected. The main problem was to match performance across emotions, because not all basic emotions were equally easy to perceive, even though we started from equivalent video stimuli (see also Swain et al., 2022). Another challenge was finding good images of different actors that were gender-balanced (four different male actors and four different female actors per emotion).

In the end, we had to settle for eight pictures per emotion, bringing the total to 48 instead of the 72 we were hoping for,^1^ because there were not enough easy items for some emotions and not enough difficult items for others. The number of items in SEE-48 is sufficient for a reliable measure of emotion recognition, but perhaps not for researchers who want to study individual emotions. For anger, happiness and surprise, additional stimuli from SEE1 and SEE2 can be chosen if needed. For the other emotions, this will be difficult. Another option could be to abandon the gender balance criterion.

Test construction was helped by the observation that emotion recognition seems to be a robust ability, affecting performance in SEE as much as in RMET and STEU (see Table 4). The latter is interesting because there is a big difference between seeing basic emotions in the eyes and knowing what emotion is felt in verbally depicted situations. So, the SEE test not only measures a low-level, perceptual skill (recognizing emotions from eyes) but also a broader ability related to emotional intelligence (Simonet et al., 2021). The correlations between SEE, RMET and STEU are in line with other studies showing good correlations between performance-based emotion recognition tests (Bryan & Mayer, 2021; Schlegel et al., 2019). They confirm that all these tests measure a common skill, while at the same time, having unique components (otherwise correlations between tests would be as high as test reliability). Campbell and Fiske (1959) already argued that each test or task used for measurement purposes is a combination of traits and measurement procedures, and that true understanding of a specific trait arises only when researchers are able to examine convergent performance from different tests expected to measure the same trait in different ways.

There are several reasons why we see the SEE-48 test as a good addition to the existing tests. First, the test scores well on reliability (Olderbak et al., 2021). Second, the test makes minimal use of language (only the names of the six basic emotions need to be known) and this makes the test useful for people with a small vocabulary or who have little experience with the test language (e.g., second-language speakers). Third, results show that test scores are not significantly correlated with anxious and apathetic traits, making the test robust to the influence of such factors (at least in university students). Fourth, the SEE-48 test provides high-graphic-quality matched stimuli for six basic emotions and actor’s gender, which opens up possibilities for testing differences in emotion perception across groups. Fifth, it allows to study the ability to recognize emotion in healthy participants avoiding ceiling effects. Finally, if a study will include enough participants, it will even become possible to analyze complete confusion matrices between the six emotions (see Barhoom et al., 2021, for methods to use).

## Availability

A test for research and assessment in clinical settings is only useful if it is easily and freely available. This allowed us to use the RAVDESS database (Livingstone & Russo, 2018) to create our own test.

To encourage further cumulative research, we are making the SEE-48 test available at https://osf.io/ed7f6/. The repository contains the selected eye stimuli, the full faces from which they were cut (for those who want to change aspects of the stimuli or compare the eye stimuli with masked faces), performance indices, and a file to present the tests in Qualtrics. Interested readers can also try out the test at https://ugent.qualtrics.com/jfe/form/SV_emuHopiA6Cn7IUe. In addition, we provide all the information from Studies 1 and 2 so that colleagues can select more stimuli if needed. The number of stimuli in the SEE-48 test could be further reduced if individual emotions are not important, if a reliability lower than 0.8 is sufficient, or if the test turns out to be too difficult for a particular population (in which case the most difficult items can be dropped). However, for the time being we believe that the SEE-48 test is short enough (usually about five minutes) to be administered in total. Some redundancy is good to make a test robust.

## Data Availability

All data, figures, and stimulus materials are available at https://osf.io/ed7f6/.

## Abbreviations

DAS: Dimensional apathy scale
RMET: Reading the mind in the eyes test
SEE: Seeing emotions in the eyes
SEE-48: Seeing emotions in the eyes test with 48 questions
STAI: State-Trait Anxiety Inventory
STEU: The situational test of emotion understanding

## Footnotes

1 Having 72 items would have been better, because then we had 12 items per emotion, giving more power to tests of a specific emotion, or making it possible to create two parallel versions of the test.
